# Full endoscopic laminotomy decompression versus anterior cervical discectomy and fusion for the treatment of single-segment cervical spinal stenosis: a retrospective, propensity score-matched study

**DOI:** 10.1186/s13018-024-04710-2

**Published:** 2024-04-05

**Authors:** Tusheng Li, Jie Huang, Hanshuo Zhang, Zhengcao Lu, Jiang Liu, Yu Ding

**Affiliations:** 1 Orthopedics of TCM Senior Department, The Sixth Medical Center of PLA General Hospital, 6 Fucheng Road, Haidian District, Beijing, 100048 People’s Republic of China; 2 The Second School of Clinical Medicine, Southern Medical University, Guangzhou, People’s Republic of China; 3 Department of Orthopaedics, School of Medicine, South China University of Technology, Guangzhou, People’s Republic of China; 4 Navy Clinical College, The Fifth School of Clinical Medicine, Anhui Medical University, Hefei, People’s Republic of China

**Keywords:** Cervical spinal stenosis, Spinal endoscopy, Laminotomy, Anterior cervical discectomy and fusion, Propensity score

## Abstract

**Objective:**

Anterior cervical discectomy and fusion (ACDF) is the standard procedure for the treatment of cervical spinal stenosis (CSS), but complications such as adjacent segment degeneration can seriously affect the long-term efficacy. Currently, posterior endoscopic surgery has been increasingly used in the clinical treatment of CSS. The aim of this study was to compare the clinical outcomes of single-segment CSS patients who underwent full endoscopic laminotomy decompression or ACDF.

**Methods:**

138 CSS patients who met the inclusion criteria from June 2018 to August 2020 were retrospectively analyzed and divided into endoscopic and ACDF groups. The propensity score matching (PSM) method was used to adjust the imbalanced confounding variables between the groups. Then, perioperative data were recorded and clinical outcomes were compared, including functional scores and imaging data. Functional scores included Visual Analog Scale of Arms (A-VAS) and Neck pain (N-VAS), Japanese Orthopedic Association score (JOA), Neck Disability Index (NDI), and imaging data included Disc Height Index (DHI), Cervical range of motion (ROM), and Ratio of grey scale (RVG).

**Results:**

After PSM, 84 patients were included in the study and followed for 24–30 months. The endoscopic group was significantly superior to the ACDF group in terms of operative time, intraoperative blood loss, incision length, and hospital stay (*P* < 0.001). Postoperative N-VAS, A-VAS, JOA, and NDI were significantly improved in both groups compared with the preoperative period (*P* < 0.001), and the endoscopic group showed better improvement at 7 days postoperatively (*P* < 0.05). The ROM changes of adjacent segments were significantly larger in the ACDF group at 12 months postoperatively and at the last follow-up (*P* < 0.05). The RVG of adjacent segments showed a decreasing trend, and the decrease was more marked in the ACDF group at last follow-up (*P* < 0.05). According to the modified MacNab criteria, the excellent and good rates in the endoscopic group and ACDF group were 90.48% and 88.10%, respectively, with no statistically significant difference (*P* > 0.05).

**Conclusion:**

Full endoscopic laminotomy decompression is demonstrated to be an efficacious alternative technique to traditional ACDF for the treatment of single-segment CSS, with the advantages of less trauma, faster recovery, and less impact on cervical spine kinematics and adjacent segmental degeneration.

## Introduction

Cervical spinal stenosis (CSS) is a common clinical spinal disorder with a high prevalence in the aging population [1]. The reduction in the effective volume of the cervical spinal canal can be due to factors such as cervical disc herniation, hypertrophy of the ligamentum flavum, ossification of the posterior longitudinal ligament, and degeneration of the facet joints, resulting in compression of the spinal cord and nerve roots, and producing symptoms of neurological dysfunction [2, 3]. The clinical manifestations of CSS patients principally include neck-shoulder pain and weakness or sensory loss, and lower limb numbness and weakness [4, 5]. In severe cases, urinary and rectal sphincter dysfunction and quadriplegia may occur [5]. For patients with mild symptoms, conservative treatment (neck immobilization, physiotherapy, medication, etc.) may provide symptomatic relief. However, for patients with progressive exacerbation of symptoms, surgery is required to prevent the progression of neurological deterioration [1, 6, 7].

Surgical approaches for CSS patients mainly include traditional open surgery and minimally invasive surgery. Anterior cervical discectomy and fusion (ACDF) is a classic open surgical procedure for the treatment of CSS, with the advantages of adequate decompression of nerves, reestablishment of cervical stability and restoration of cervical physiological lordosis [8, 9]. However, ACDF still has some limitations including potential complications such as degeneration of adjacent segments, restriction in neck movement, and displacement of the fusion device [5, 9]. In recent years, with the continuous development and refinement of minimally invasive concepts, spinal endoscopic surgery has been increasingly used in the clinical treatment of CSS due to its advantages of less trauma, faster recovery, and fewer complications, aiming to reduce soft tissue damage and achieve the same therapeutic effect as open surgery [6, 10, 11]. Nevertheless, the feasibility, indications, and clinical efficacy of spinal endoscopic surgery still need to be further investigated and clarified due to the limited literature available. Our team has previously applied full endoscopic laminotomy decompression to treat lumbar spinal stenosis with satisfactory clinical efficacy [12], and our guiding hypothesis was that this approach could also be applied to CSS with good clinical outcomes. In this study, full endoscopic laminotomy decompression or ACDF was performed for the treatment of CSS, with the aim of evaluating the clinical safety and superiority of the two surgical approaches.

## Materials and methods

### Study design and patients

This study was a single-center clinical retrospective study approved by the Ethics Committee (No. HZKY-PJ-2023-40), and all patients signed a written informed consent before treatment. From June 2018 to August 2020, 138 patients with single-segment CSS were included in the study according to the inclusion and exclusion criteria (Table 1), of which 62 were treated with full endoscopic laminotomy decompression (endoscopic group) and 76 were treated with ACDF (ACDF group). Considering the non-randomized nature of the study and various factors that could influence the outcomes, we designed a propensity score matching (PSM) cohort (caliper value set at 0.02) to balance the impact of confounding factors when comparing clinical outcomes between the two groups. The propensity score for each patient was calculated as a probability using a logistic regression model, including all covariates considered clinically important and potentially affecting clinical outcomes: (1) age, (2) body mass index (BMI), (3) gender, (4) disease duration, (5) smoking history, (6) medical history, (7) operative segment, and (8) pathological type.

**Table 1:** Inclusion and exclusion criteria

Inclusion criteria	Single-segment cervical spinal stenosis
	Imaging showing spinal cord and/or nerve root compression
	Failure of conservative treatment
	Good condition and can tolerate general anesthesia surgery
Exclusion criteria	Cervical spine deformities and infection
	Developmental cervical spinal stenosis
	Severe osteoporosis
	Cervical spine tumors and other malignant tumors
	Previous history of cervical spine injury or surgery
	Osteoarthritis diseases, such as rheumatoid arthritis, ankylosing spondylitis

### Surgical methods

All procedures were performed by the same surgical group with extensive clinical experience. The following techniques were used:

### Endoscopic approach

**Fig. 1:**
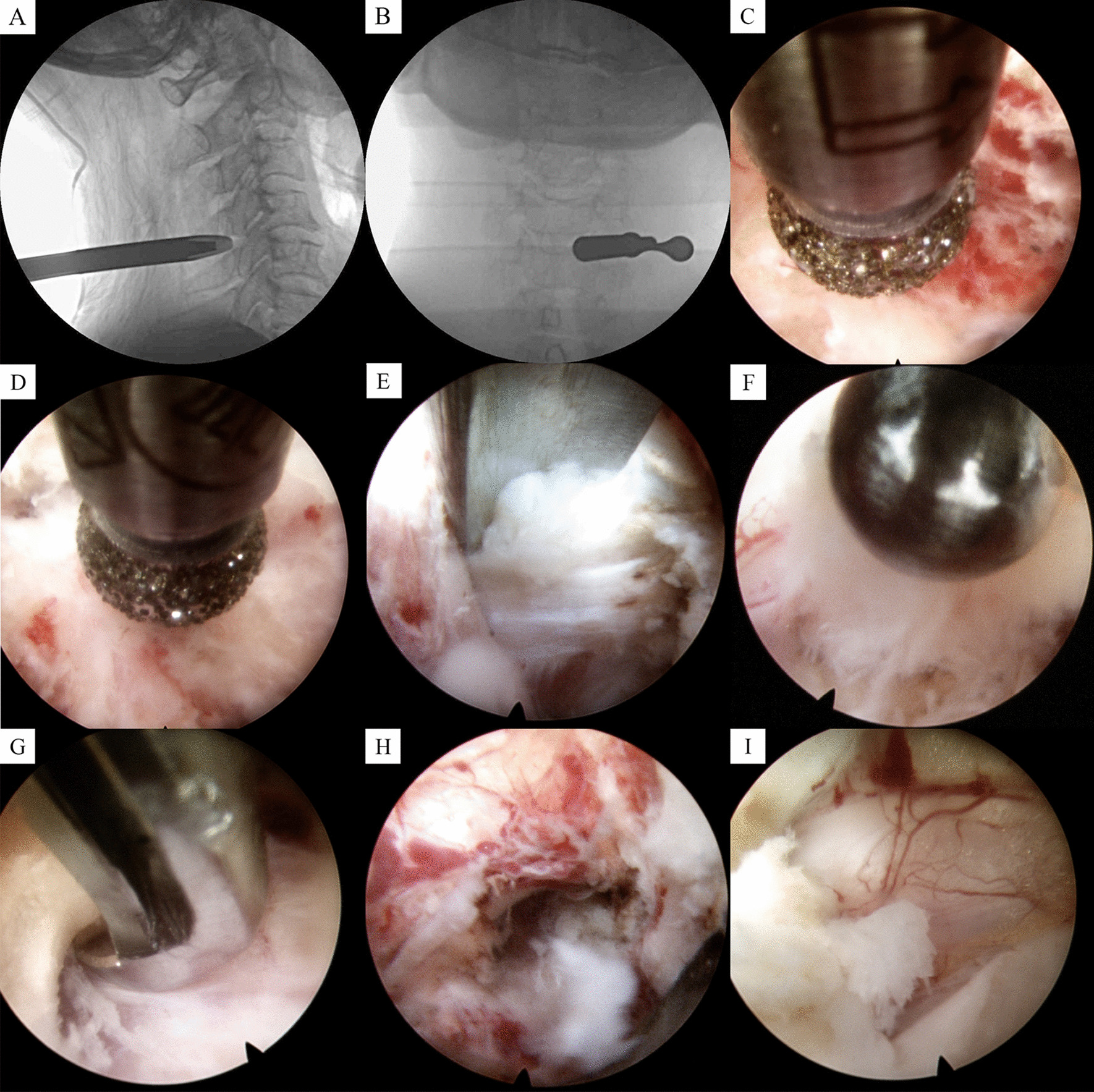
Endoscopy diagrams. **A**, **B** Intraoperative working cannula placement; **C** Removal of the vertebral plates by grinding drills; **D** Removal of the inner edge of the facet joints; **E** Herniated disc tissue compressing nerve root; **F** Removal of herniated disc tissue; **G** Removal of ligamentum flavum; **H** Nerve root decompression; **I** Dural sac decompression

**Fig. 2:**
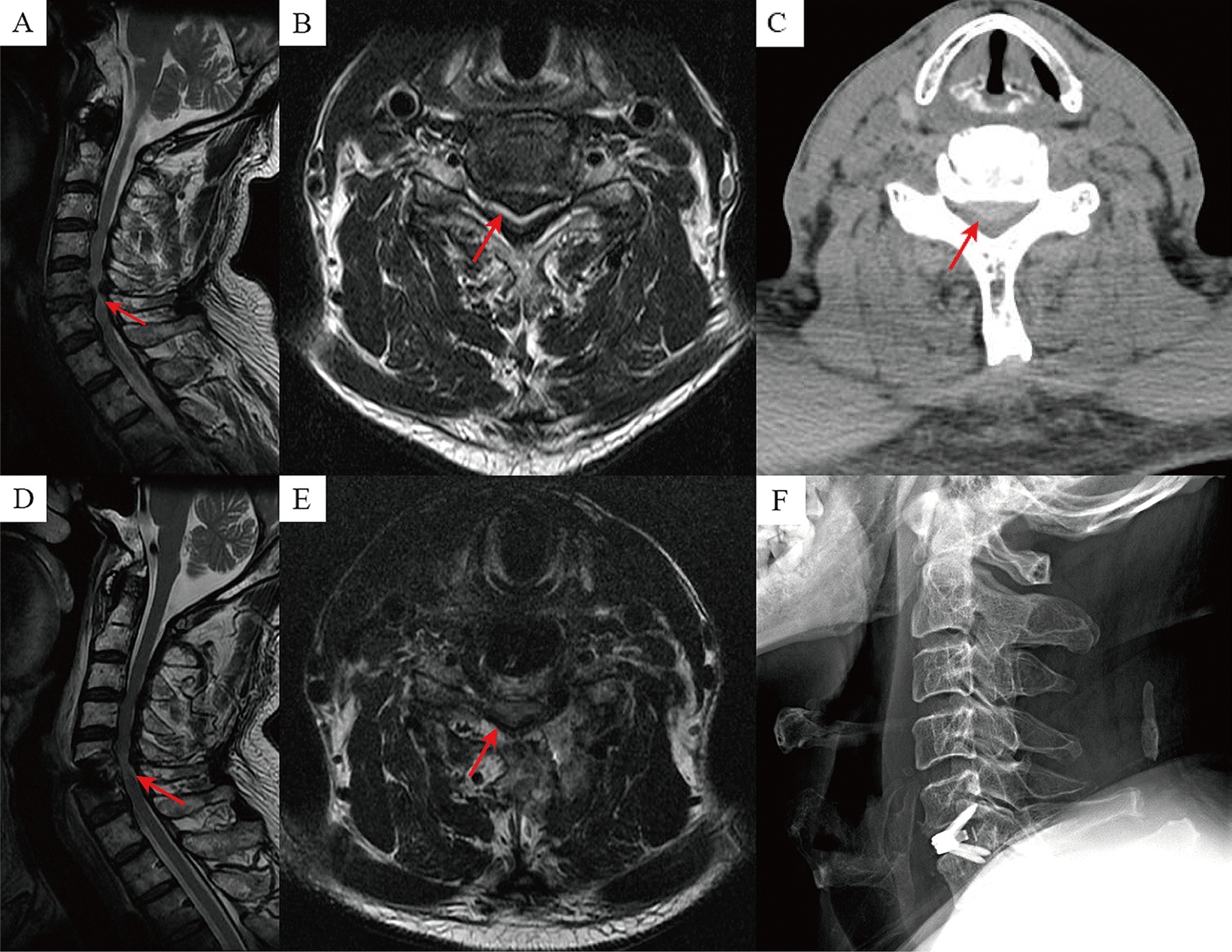
Images from a patient treated with ACDF. **A**–**C** Preoperative MRI and CT showed spinal stenosis of C5–6 segment, and compression of the spinal cord; **D**, **E** Postoperative MRI showed adequate decompression of the spinal canal and spinal cord; **F** Postoperative interbody fusion with internal fixation

The patient was placed in a prone position with the head fixed and the neck slightly flexed. C-arm X-ray fluoroscopy was used to locate the puncture point of the surgical segment, which was the lateral angle of the intervertebral plate space 1–2 cm adjacent to the spinous process (vertebral plate intersection “V” point). The needle was introduced at the puncture point, and fluoroscopy confirmed that the tip of the needle was located near the posterolateral corner of the intervertebral space, in contact with the posterior surface of the facet joint. A 7–10 mm surgical incision was made, and the surgical channel was expanded to the bony surface of the vertebral plate using a soft-tissue dilator tube. Then, the spinal endoscopic light source and imaging system (TH8700-030L, Cisco, Germany) was connected, and a 6.5-mm endoscope was placed. Under endoscopic control, a grinding drill was used to remove part of the outer layers of the upper and lower vertebral plates and the inner edge of the facet joints from the “V” point outward. The surgical segment and the boundary of the decompression range were again confirmed by fluoroscopy. The inner layers of the vertebral plates were removed and a conical fenestration was created anteriorly until reaching the connection between the facet joint and the vertebral body (referred to as the “bell mouth” fenestration). During the process of fenestration, the ligamentum flavum was gradually removed, and the decompression range of the intervertebral plate, facet joints, and root of the spinous process was expanded. Endoscopic visualization clearly demonstrated the nerve roots, dural sac and intervertebral disc tissue. The compression-causing areas such as the anterior side of the nerve root and the ventral side of the dural sac were then decompressed to achieve adequate space of the spinal canal, and if necessary, protruding disc tissue and posterior osteophyte were excised. For cases that suffer from ventral disc herniation of the dural sac and/or ossification of the posterior longitudinal ligament, the facet joints should be resected outward more, and a portion of the pedicle and the vertebral body could be removed. Subsequently, the compressive materials in front of the spinal cord were removed from a posterolateral to anteromedial approach through the fenestration pathway. For cases with bilateral spinal stenosis, full decompression was performed firstly on the side where the spinal stenosis was relatively severe and/or primarily responsible for symptoms and signs. Then, the lamina was progressively resected inward to the bone contralateral to the root of the spinous process, and the contralateral spinal canal could be adequately decompressed using the “over-top” technique. If necessary, bilateral decompression was chosen. Decompression was considered successful if the nerve root tension decreased, there was no compressive tissue around the nerve, and the nerve root and dural sac showed autonomous pulsation. After adequate hemostasis, the spinal endoscope was withdrawn and the incision closed. Endoscopy diagrams and representative cases are shown in Figs. 1 and 2, respectively.

### ACDF approach

**Fig. 3:**
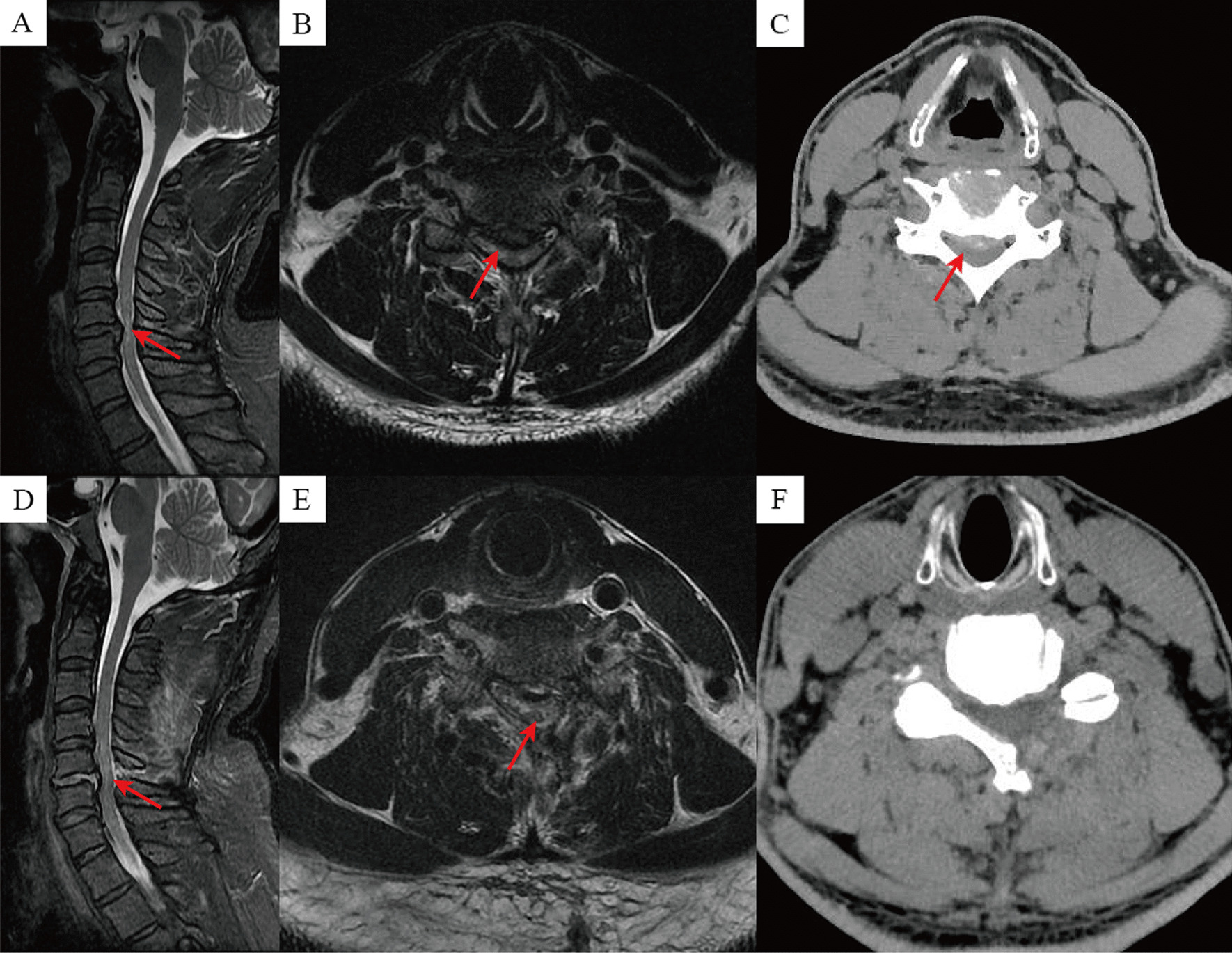
Images from a patient treated with full endoscopic laminotomy decompression. **A**–**C** Preoperative MRI and CT showed spinal stenosis of C5–6 segment, and compression of the spinal cord; **D**–**F** Postoperative MRI and CT showed adequate decompression of the spinal canal and spinal cord

The surgical procedure was performed using the standard Smith-Robinson approach. A right-sided transverse or longitudinal incision was made on the anterior neck to fully expose the anterior edge of the vertebral body, and to excise the anterior longitudinal ligament of the surgical segment. The herniated disc tissue was scraped away using a curette and the intervertebral space was propped open using a distractor. The posterior longitudinal ligament was resected if necessary, and for posterior-lateral decompression, the upper and lower cartilaginous endplates were scraped and the hyperplastic posterior facet joints was removed using a vertebral plate bone forceps to achieve adequate decompression of the spinal canal. The intervertebral space was flushed, and the appropriate model of Zero-P interbody fusion device (PEEK Materials, AO Company, Switzerland) was selected for placement into the intervertebral space and fixed with screws. After adequate hemostasis, a drainage tube was placed and the incision was closed. Representative cases are shown in Fig. 3.

All patients received routine perioperative management such as nutritional support, nerve protection, and infection prevention. After discharge, patients were instructed to wear a neck brace and restrict cervical spine movement for 4–6 weeks.

### Data collection and measurements

Preoperative baseline and perioperative data were collected from all successfully matched CSS patients. Perioperative data included operative time, intraoperative blood loss, incision length, and hospital stay. Patients were followed up regularly postoperatively through phone calls and/or emails to record their clinical functional scores, imaging data, and the occurrence of complications.

### Clinical evaluation

Patient clinical pain was assessed using Visual Analog Scale of Arms (A-VAS) and Neck (N-VAS), and cervical spine dysfunction was assessed using Japanese Orthopedic Association score (JOA) and Neck Disability Index (NDI). Additionally, we used the minimal clinically important difference (MCID) to evaluate the clinical significance of changes in N-VAS, A-VAS, JOA and NDI. MCID value changes of ≥ 2.6, ≥ 4.1, ≥ 2.5, and ≥ 17.3% were used for N-VAS, A-VAS, JOA, and NDI, respectively [13, 14]. At the last follow-up, patient satisfaction with the procedure was assessed using the modified MacNab criteria [15].

### Imaging measurements

Patients in both groups underwent cervical spine X-rays including lateral, flexion and extension positions as well as magnetic resonance imaging (MRI) scans. Imaging data were measured using Image Viewer or AnyPacs software installed on workstations in DICOM or JPG format. All imaging data were measured three times by three independent evaluators and averaged. The symbol “+” indicates cervical kyphosis, and “−” indicates cervical lordosis.

**Fig. 4:**
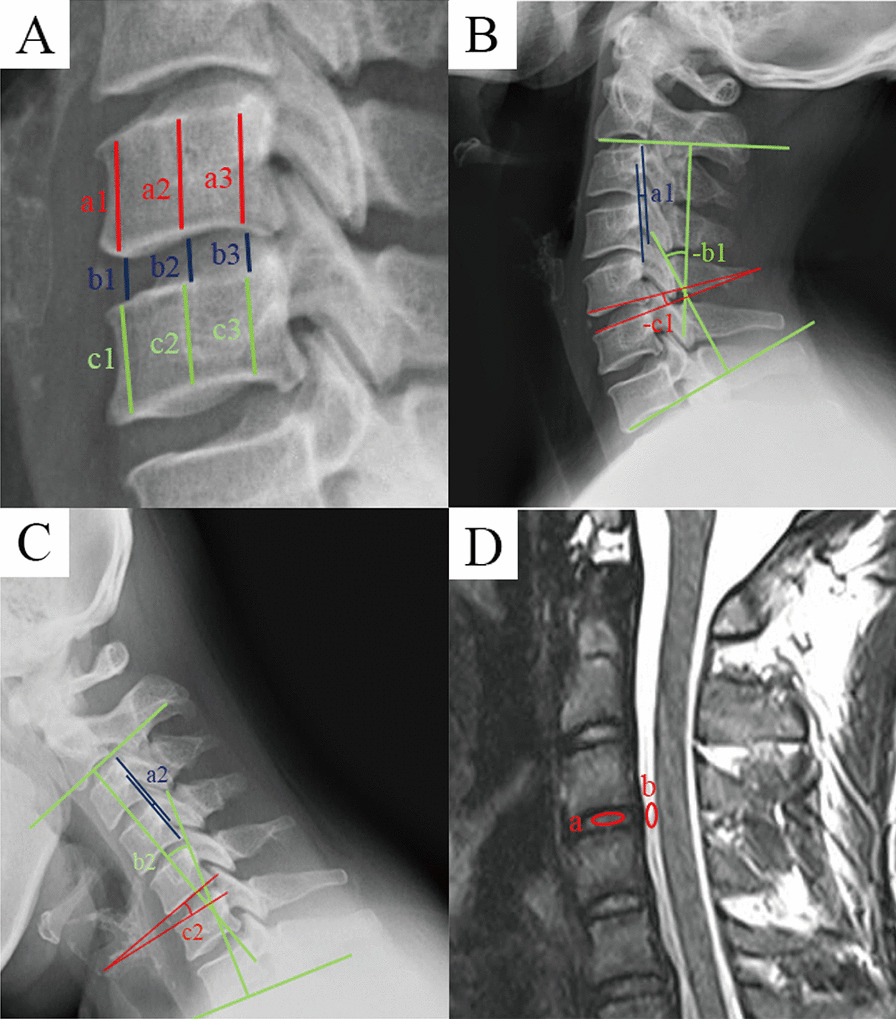
Schematic of imaging measurements. **A** Disc height index (DHI), DHI = [2(b1 + b2 + b3)]/[(a1 + a2 + a3) + (c1 + c2 + c3)] * 100%. **B**, **C** Schematic diagram of sagittal translation (ST) and the range of motion (ROM). **B** The measurements in hyperextension position. **C** The measurements in hyperflexion position. ST = a1–a2, GROM = b2- (-b1), Adjacent segment ROM = c2- (-c1). **D** Ratio value of the greyscale (RVG). Midsagittal T2-weighted images were chosen, and RVG was the greyscale of discs (a) normalized against the greyscale of cerebrospinal fluid at the same level (b)

1. *Disc Height Index (DHI)* Following the methodology of previous studies [16], the DHI assessed changes in disc height at different follow-up time points. The anterior, middle, and posterior heights of the upper and lower vertebral body and intervertebral discs were measured on lateral X-ray of the cervical spine. DHI was calculated as the ratio of the sum of intervertebral disc heights to the sum of upper and lower vertebral body heights: DHI = [2(b1 + b2 + b3)]/[(a1 + a2 + a3) + (c1 + c2 + c3)] *100%, as shown in Fig. 4A.
2. *Sagittal translation (ST)* The principle of translational instability (TI) was used to assess the stability of the cervical spine after full endoscopic laminotomy decompression for CSS [17]. The translational distance of the posterior edge of the vertebral body of adjacent cervical segments was measured on radiographs in the hyperextension and hyperflexion positions, subtracted in the same direction and added in the opposite direction. Cervical segmental instability was defined when the ST was ≥ 3 mm [18], as shown in Fig. 4B and C.
3. *Range of motion (ROM)* The overall curvature of the cervical spine on hyperextension and hyperflexion positions was measured using Cobb’s method, and the difference between the two was the global range of motion (GROM) [19]. Segmental angle was formed by the line connecting the inferior endplate of the superior vertebra to the superior endplate of the inferior vertebra. ROM = angle in hyperextension position—angle in hyperflexion position. The superior adjacent segment ROM (SROM) and the inferior adjacent segment ROM (IROM) were measured respectively using this method, as shown in Fig. 4B and C.
4. *The ratio value of the greyscale (RVG)* Based on the modified Schneiderman method, RVG was used to assess the water content of intervertebral discs [20]. MRI median sagittal T2-weighted images were imported into Photoshop software (Adobe Photoshop version 2023), and the average greyscale values of the discs and cerebrospinal fluid were measured at the same segment. RVG was calculated as the ratio of the two: RVG = (average grayscale value of the intervertebral disc/average grayscale value of cerebrospinal fluid) * 100%. The superior adjacent segment RVG (SRVG) and the inferior adjacent segment RVG (IRVG) were measured respectively using this method, as shown in Fig. 4D.

### Statistical analysis

For normally distributed continuous data, Student’s t-test analysis was used and expressed as means ± standard deviation (SD). Within-group comparisons at different time points were performed using repeated measures analysis of variance. For non-normally distributed data, non-parametric tests were applied. To assess the balance between groups, the standardized mean difference (SMD) was calculated to represent the intergroup balance for a given covariate [21]. SMD is not influenced by sample size and allows for the comparison of relative balance between variables. According to Cohen’s criteria, an SMD ≤ 0.2 indicates a small difference for the covariate [21]. Categorical data were presented as frequencies and percentages and compared using the chi-square test. A *P*-value < 0.05 was considered statistically significant for differences between the two groups. All statistical analyses were conducted using SPSS version 25 (IBM SPSS Statistics for Windows, Version 25.0. Armonk, NY: IBM Corp.).

## Results

### Baseline characteristics before and after PSM

Based on the inclusion and exclusion criteria, 138 CSS patients were included in the study, 62 in the endoscopy group and 76 in the ACDF group. The baseline characteristics of the two groups before PSM are shown in Table 2. Covariates with SMD ≤ 0.2 and *P* > 0.05 were considered balanced and comparable between the two groups. However, two covariates were seen to be unbalanced in Table 2, including age (SMD = 0.352, *P* = 0.039) and disease duration (SMD = 0.434, *P* = 0.011). After PSM, 84 CSS patients remained in the study, and the baseline characteristics of the two groups are shown in Table 3, where it could be observed that all covariates were well balanced and comparable. All patients were followed for at least 2 years (range 24–30 months). The mean follow-up time was 26.10 ± 1.85 months for the endoscopic group and 25.98 ± 1.65 months for the ACDF group, with no significant difference between the groups (*P* = 0.884).

**Table 2:** Demographic characteristics before propensity score matching

Demographics	Endoscopy group (n = 62)	ACDF group (n = 76)	SMD	*P* value
Age (years)	63.44 ± 8.38	66.47 ± 8.59	**0.352**	**0.039**
BMI (kg/m^2^)	23.65 ± 2.95	23.74 ± 2.73	0.032	0.856
Gender, n (%)			0.009	0.960
Male	34 (54.8)	42 (55.3)		
Female	28 (45.2)	34 (44.7)		
Disease duration (months)	20.98 ± 8.27	24.51 ± 7.72	**0.434**	**0.011**
Smoking, n (%)	20 (32.3)	25 (32.9)	0.014	0.937
Medical history, n (%)				
Hypertension	19 (30.6)	24 (31.6)	0.020	0.906
Diabetes	20 (32.3)	25 (32.9)	0.014	0.937
Operative segment			0.000	0.999
C3–4	6 (9.7)	7 (9.2)		
C4–5	15 (24.2)	18 (23.7)		
C5–6	23 (37.1)	29 (38.2)		
C6–7	18 (29.0)	22 (28.9)		
Pathological type, n (%)			0.007	0.928
Myelopathy	41 (66.1)	48 (63.2)		
Myelo-radiculopathy	19 (30.6)	25 (32.9)		
Radiculopathy	2 (3.2)	3 (3.9)		

Bolding is to indicate SMD > 0.2 or P ≤ 0.05, which means that the corresponding confounders are not balanced between the two groups

Continuous data are presented as mean ± SD; categorical data are presented as n (%); P < 0.05 considered significant; *ACDF* anterior cervical discectomy and fusion; *BMI* body mass index; *SMD* standardized mean difference; absolute value of SMD > 0.2 considered unbalanced

**Table 3:** Demographic characteristics after propensity score matching

Demographics	Endoscopy group (n = 42)	ACDF group (n = 42)	SMD	*P* value
Age (years)	64.43 ± 7.84	64.31 ± 9.46	0.014	0.950
BMI (kg/m^2^)	23.31 ± 2.82	23.38 ± 2.59	0.026	0.907
Gender, n (%)			0.095	0.662
Male	23 (54.8)	21 (50.0)		
Female	19 (45.2)	21 (50.0)		
Disease duration (months)	22.60 ± 8.48	22.88 ± 7.40	0.035	0.870
Smoking, n (%)	16 (38.1)	13 (31.0)	0.149	0.491
Medical history, n (%)				
Hypertension	14 (33.3)	12 (28.6)	0.102	0.637
Diabetes	17 (40.5)	13 (31.0)	0.198	0.362
Operative segment			0.018	0.712
C3–4	5 (11.9)	3 (7.1)		
C4–5	8 (19.0)	12 (28.6)		
C5–6	17 (40.5)	16 (38.1)		
C6–7	12 (28.6)	11 (26.2)		
Pathological type, n (%)			0.048	0.325
Myelopathy	27 (64.3)	30 (71.4)		
Myelo-radiculopathy	14 (33.3)	9 (21.4)		
Radiculopathy	1 (2.4)	3 (7.1)		

Continuous data are presented as mean ± SD; categorical data are presented as n (%);* P* < 0.05 considered significant; *ACDF* anterior cervical discectomy and fusion, *BMI* body mass index, *SMD* standardized mean difference; absolute value of SMD > 0.2 considered unbalanced

### Perioperative data

The perioperative data for both groups are presented in Table 4. In the endoscopic group, the average operative time was 85.43 ± 5.16 min, intraoperative blood loss was 8.29 ± 2.68 ml, incision length was 0.83 ± 0.12 cm, and hospital stay was 6.14 ± 0.87 days, whereas in the ACDF group, the corresponding values were 98.48 ± 7.84 min, 50.40 ± 4.46 ml, 3.68 ± 0.29 cm, and 8.07 ± 0.84 days, respectively. These superior results in the endoscopic group were all significantly better than the ACDF group (*P* < 0.001).

**Table 4:** The perioperative data between two groups

	Endoscopy group (n = 42)	ACDF group (n = 42)	*P* value
Operative time (min)	85.43 ± 5.16	98.48 ± 7.84	< 0.001
Intraoperative blood loss (ml)	8.29 ± 2.68	50.40 ± 4.46	< 0.001
Total length of incision (cm)	0.83 ± 0.12	3.68 ± 0.29	< 0.001
Hospital stay (d)	6.14 ± 0.87	8.07 ± 0.84	< 0.001

Continuous data are presented as mean ± SD; *P* < 0.05 considered significant; *ACDF* anterior cervical discectomy and fusion; *min* minutes; *ml* milliliters; *d* days; *cm* centimetres

### Clinical outcomes

**Fig. 5:**
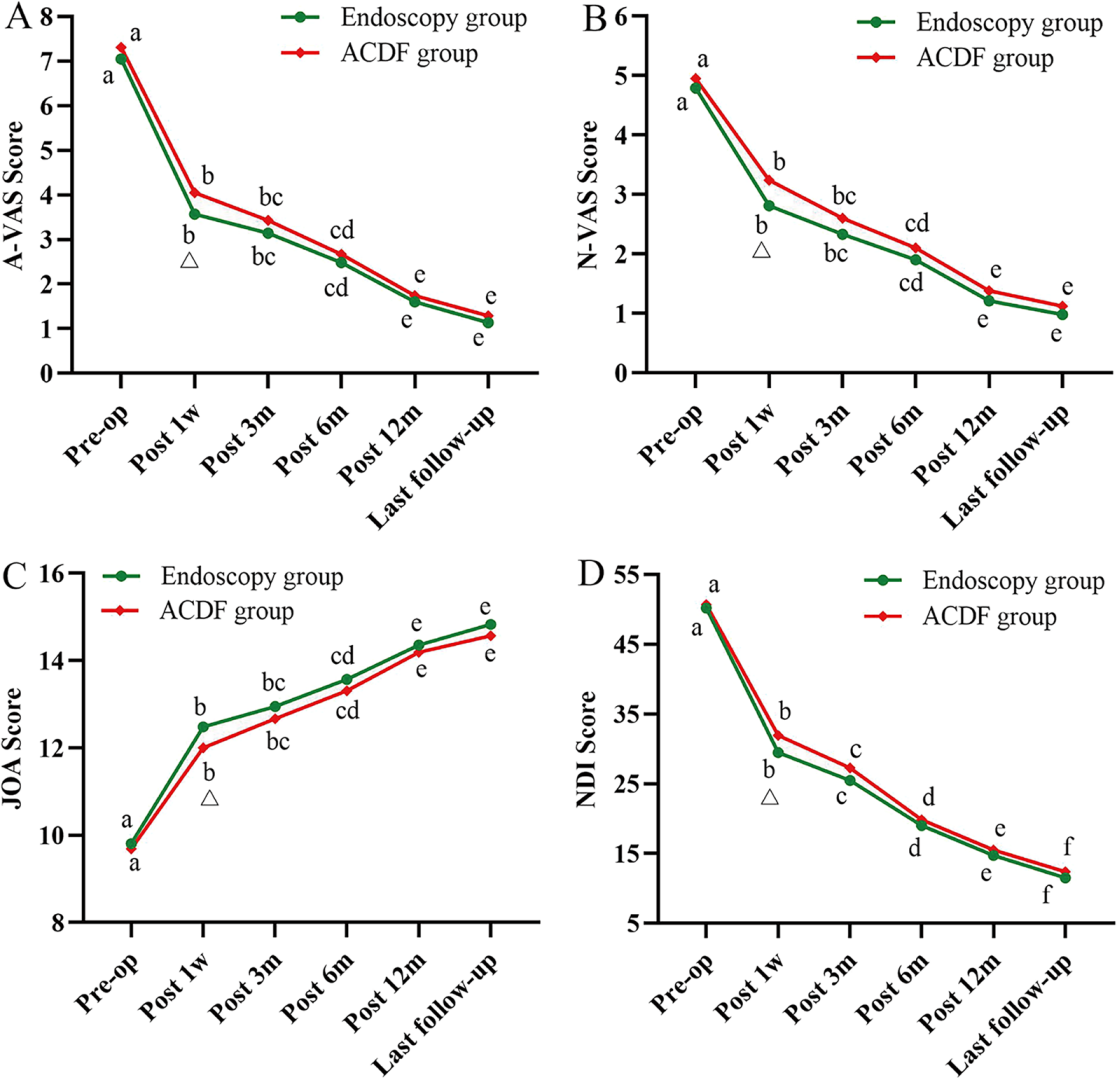
Results of clinical efficacy of functional scores. **A** Changes in A-VAS scores over time. **B** Changes in N-VAS scores over time. **C** Changes in JOA scores over time. **D** Changes in NDI scores over time. A-VAS, Arm Visual Analog Scale; N-VAS, Neck Visual Analog Scale; JOA, Japanese Orthopedic Association; NDI, Neck Disability Index. a-f indicate the letter labelling of the time point difference (comparison within the group), if 2 time points have the same letter, there is no significant difference between the 2 time points (*P* > 0.05); otherwise, different letters at 2-time points mean the difference is significant (*P* ≤ 0.05). Δ represents a significant difference between the two groups

Visual Analog Scale (VAS) of Pain: The mean A-VAS scores in the endoscopic and ACDF groups decreased from 7.05 ± 0.94 and 7.31 ± 0.87 before operation to 3.57 ± 0.83 and 4.05 ± 0.94, respectively, at 7 days postoperatively (*P* = 0.016, *P* = 0.044); 3.14 ± 0.68 and 3.43 ± 0.80 at 3 months postoperatively (*P* < 0.001); 2.48 ± 0.92 and 2.67 ± 0.98 at 6 months postoperatively (*P* < 0.001); 1.60 ± 0.83 and 1.74 ± 0.77 (*P* < 0.001) at 12 months postoperatively; and 1.14 ± 0.72 and 1.29 ± 0.81 at last follow-up (*P* < 0.001). Both groups showed significant improvement in A-VAS scores postoperatively compared to preoperatively (*P* < 0.001), and the improvements met the clinical significance criteria for MCID. At 7 days postoperatively, the improvement in A-VAS score was better in the endoscopic group than the ACDF group (*P* = 0.022) (Fig. 5A).

The mean N-VAS scores showed nearly the same trend as the mean A-VAS scores in both groups (Fig. 5B).

Japanese Orthopedic Association score (JOA): The mean JOA scores in the endoscopic and ACDF groups significantly improved from 9.81 ± 0.67 and 9.69 ± 0.75 preoperatively to 12.48 ± 0.94 and 12.00 ± 1.01 at 7 days postoperatively (*P* = 0.013, *P* = 0.048); 12.95 ± 0.76 and 12.67 ± 0.90 at 3 months postoperatively (*P* < 0.001); 13.57 ± 0.89 and 13.31 ± 1.05 at 6 months postoperatively (*P* < 0.001); 14.36 ± 0.88 and 14.19 ± 0.99 at 12 months postoperatively (*P* < 0.001); and 14.83 ± 0.96 and 14.57 ± 1.02 at last follow-up (*P* < 0.001). Postoperative JOA scores improved significantly in both groups compared with the preoperative period and met the clinical significance criteria of MCID. At 7 days postoperatively, the improvement in JOA score was better in the endoscopic group than the ACDF group (*P* = 0.035) (Fig. 5C).

Neck Disability Index (NDI): The mean NDI scores in the endoscopic and ACDF groups decreased from 50.19 ± 4.07 and 50.67 ± 5.24 preoperatively to 29.48 ± 5.32 and 31.95 ± 5.77 at 7 days postoperatively (*P* < 0.001); 25.48 ± 4.61 and 27.29 ± 4.82 at 3 months postoperatively (*P* < 0.001); 19.00 ± 4.22 and 19.86 ± 4.68 at 6 months postoperatively (*P* < 0.001); 14.71 ± 3.72 and 15.52 ± 4.14 at 12 months postoperatively; and 11.52 ± 3.53 and 12.38 ± 4.03 at last follow-up (*P* < 0.001). Both groups showed significant improvement in NDI scores postoperatively compared to preoperative values, and the improvements met the clinical significance criteria for MCID. At 7 days postoperatively, the improvement in NDI score was better in the endoscopic group than in the ACDF group (*P* = 0.044) (Fig. 5D).

At the last follow-up, according to the modified MacNab criteria, there were 20 cases of excellent, 18 cases of good, 4 cases of fair, and 0 cases of poor in the endoscopic group, with an excellence and good rate of 90.48%; there were 19 cases of excellent, 18 cases of good, 4 cases of fair, and 1 case of poor in the ACDF group, with an excellence and good rate of 88.10%. There was no statistically significant difference in the comparison of the excellent and good rate between the two groups (*P* = 0.795) (Table 5).

**Table 5:** Comparison of MacNab evaluation and complications between the two groups

	Endoscopy group (n = 42)	ACDF group (n = 42)	*P* value
MacNab evaluation			0.795
Excellence	20 (47.62)	19 (45.24)	
Good	18 (42.86)	18 (42.86)	
Fair	4 (9.52)	4 (9.52)	
Poor	0 (0.00)	1 (2.38)	
Excellence/good rate	90.48%	88.10%	
Complications			0.178
AS or neurological dysfunction	1	2	
CSF leakage	2	2	
Dysphagia	0	1	
Hoarseness	0	1	
Revision	0	1	

*AS* axial symptoms, *CSF leakage* cerebrospinal fluid leakage, *ACDF* anterior cervical discectomy and fusion

### Image measurement

**Fig. 6:**
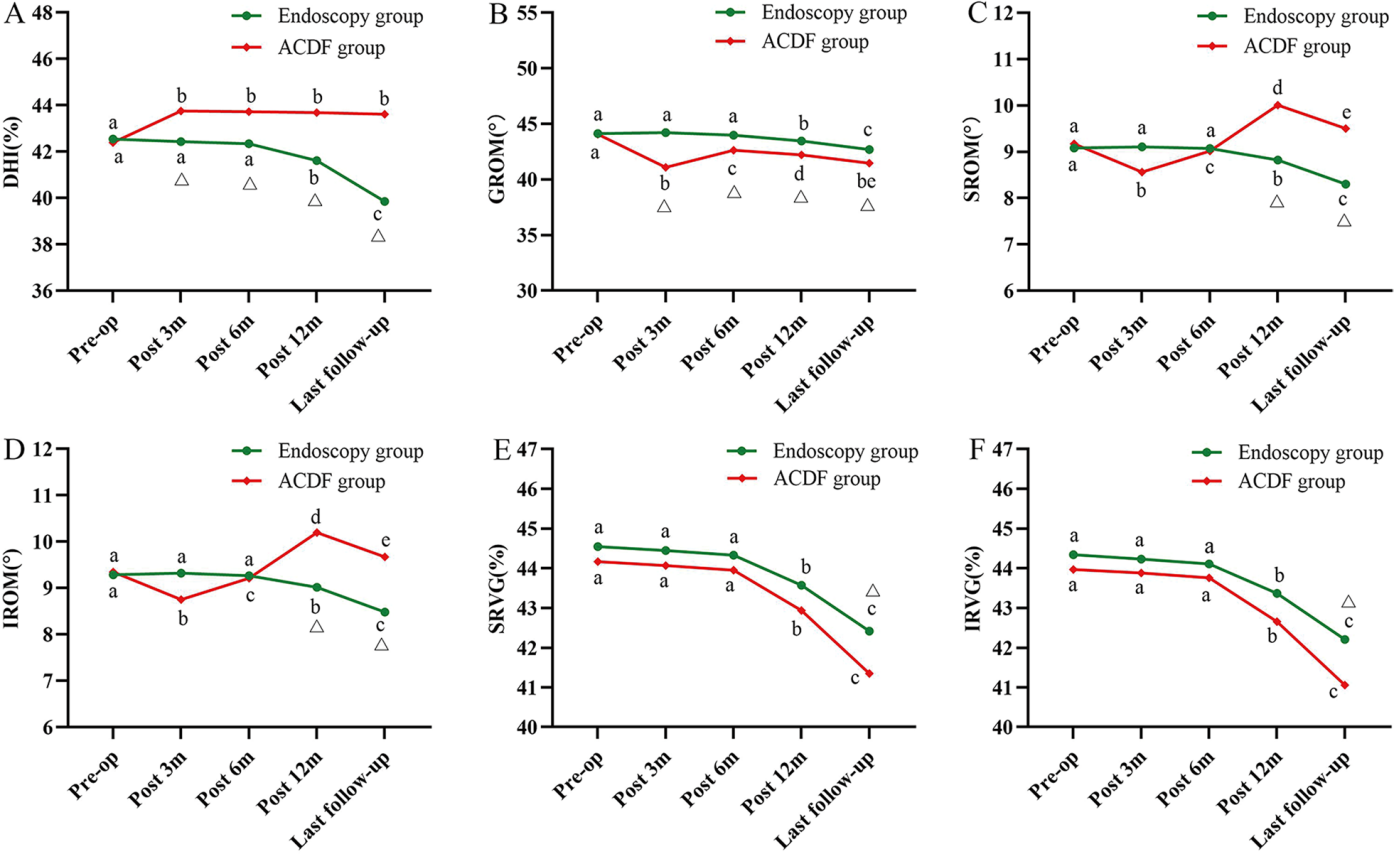
Results of imaging measurement. **A** Changes in DHI during the follow-up.** B** Changes in GROM during the follow-up. **C**, **D** Changes in ROM of the superior and inferior adjacent segments, respectively; **E**, **F** Changes in RVG of the superior and inferior adjacent segments, respectively. DHI, Disc Height Index; GROM, the global range of motion; SROM, the superior adjacent segment range of motion; IROM, the inferior adjacent segment range of motion; SRVG, the superior adjacent segment ratio value of the greyscale; IRVG, the inferior adjacent segment ratio value of the greyscale. a–e indicate the letter labelling of the time point difference (comparison within the group); if 2 time points have the same letter, there is no significant difference between the 2 time points (*P* > 0.05); otherwise, different letters at 2-time points mean the difference is significant (*P* ≤ 0.05). Δ represents a significant difference between the two groups

Disc Height Index (DHI): The mean DHI of surgical segments in the endoscopic group showed a decreasing trend from (42.55 ± 2.55) % preoperatively to (42.43 ± 2.47) % at 3 months postoperatively (*P* = 0.055); (42.34 ± 2.43) % at 6 months postoperatively (*P* = 0.053); (41.62 ± 2.33) % at 12 months postoperatively (*P* < 0.001); and (39.86 ± 2.20) % at last follow-up (*P* < 0.001). The mean DHI of surgical segments in the ACDF group increased from (42.39 ± 2.69) % before preoperatively to (43.76 ± 2.68) % at 3 months postoperatively (*P* < 0.001); and then remained essentially stable during the follow-up period. The ACDF group had a significant advantage over the endoscopic group in terms of the maintenance of DHI postoperatively (*P* < 0.05) (Fig. 6A).

Sagittal translation (ST): The mean ST of surgical segments in the endoscopic group was 1.24 ± 0.24 (mm) preoperatively; 1.26 ± 0.25 (mm) at 3 months postoperatively (*P* = 0.107); 1.28 ± 0.25 (mm) at 6 months postoperatively (*P* = 0.079); 1.34 ± 0.27 (mm) at 12 months postoperatively (*P* < 0.001); and 1.39 ± 0.27 (mm) at last follow-up (*P* < 0.001). ST was statistically different at 12 months postoperatively and at last follow-up compared with preoperatively (*P* < 0.05) (Table 6). However, all patients had a postoperative ST < 3 mm, indicating that cervical segmental stability was still well maintained after full endoscopic laminotomy decompression for CSS during the follow-up period.

**Table 6:** Imaging data of sagittal translation in the endoscopic group

Follow-up period	Sagittal translation (mm)	*P*-value compared to pre-op
Pre-op	1.24 ± 0.24 (0.85–1.88)	–
Post 3 m	1.26 ± 0.25 (0.85–1.93)	*P* = 0.107
Post 6 m	1.28 ± 0.25 (0.90–1.99)	*P* = 0.079
Post 12 m	1.34 ± 0.27 (0.95–2.05)	*P* < 0.001
Last follow-up	1.39 ± 0.27 (1.03–2.12)	*P* < 0.001

Continuous data are presented as mean ± SD; *P* < 0.05 considered significant; pre-op, preoperatively; post 3 m, 3 months postoperatively; post 6 m, 6 months postoperatively; post 12 m, 12 months postoperatively

*Range of motion (ROM)* The measurements of cervical range of motion include GROM, SROM and IROM.

The mean GROMs in the endoscopic and ACDF groups were (44.14 ± 3.13)° and (44.07 ± 3.03)° preoperatively, (44.21 ± 3.12)° and (41.09 ± 3.01)° at 3 months postoperatively (*P* = 0.072, *P* < 0.001); (43.99 ± 2.85)° and (42.64 ± 2.95)° at 6 months postoperatively (*P* = 0.824, *P* < 0.001); (43.46 ± 2.75)° and (42.22 ± 2.87)° at 12 months postoperatively (*P* < 0.001); and (42.70 ± 2.67)° and (41.47 ± 2.93)° at last follow-up (*P* < 0.001). Compared with the endoscopic group, postoperative GROM was reduced significantly in the ACDF group, and the difference between the two groups was statistically different (*P* < 0.05) (Fig. 6B).

The mean SROM in the endoscopic and ACDF groups were (9.08 ± 1.52)° and (9.17 ± 1.39)° preoperatively, (9.11 ± 1.44)° and (8.56 ± 1.37)° at 3 months postoperatively (*P* = 1.000, *P* < 0.001); (9.07 ± 1.41)° and (9.02 ± 1.43)° at 6 months postoperatively (*P* = 1.000, *P* < 0.001); (8.82 ± 1.38)° and (10.01 ± 1.46)° at 12 months postoperatively (*P* < 0.001); and (8.30 ± 1.33)° and (9.50 ± 1.41)° at last follow-up (*P* < 0.001). Compared with the endoscopic group, the SROM was significantly larger in the ACDF group at 12 months postoperatively and at the last follow-up, and the difference was statistically significant (*P* < 0.001) (Fig. 6C).

The trends of IROM and SROM were similar in the endoscopic and ACDF groups, and the difference between the two groups was statistically significant at 12 months postoperatively and at last follow-up (*P* < 0.001), as shown in Fig. 6D.

The ratio value of the greyscale (RVG): The mean SRVG in the endoscopic and ACDF groups decreased from (44.55 ± 2.48) % and (44.17 ± 2.60) % preoperatively to (44.45 ± 2.40) % and (44.07 ± 2.58) % at 3 months postoperatively (*P* = 0.177, *P* = 0.148); (44.33 ± 2.38) % and (43.95 ± 2.57) % at 6 months postoperatively (*P* = 0.106, *P* = 0.087); (43.57 ± 2.36) % and (42.94 ± 2.48) % at 12 months postoperatively (*P* < 0.001); and (42.42 ± 2.29) % and (41.35 ± 2.42) % at last follow-up (*P* < 0.001). The trend of decreasing SRVG was more pronounced in the ACDF group than in the endoscopy group at last follow-up (*P* = 0.041), as shown in Fig. 6E.

The trends of IRVG and SRVG were similar in both groups. At the last follow-up, the decreasing trend of IRVG was more pronounced in the ACDF group than in the endoscopy group (*P* = 0.032), as shown in Fig. 6F.

### Complications

Neurological dysfunction is one of the major complications in cervical spine surgery, primarily attributed to the aberrant internal milieu of the cervical spine leading to nerve injury and subsequent dysfunction of original nerve function [1, 22, 23]. Patients commonly manifest symptoms including paralysis, pain, and weakness within the innervated region at postoperative. In the endoscopic group, there was one case of neurological dysfunction with C5 nerve root palsy, and two cases of cerebrospinal fluid leakage. In the ACDF group, there were two cases of axial pain or neurological dysfunction, two cases of cerebrospinal fluid leakage, one case of dysphagia, and one case of hoarseness. All patient symptoms were satisfactorily relieved after conservative treatment, except for one patient in the ACDF group underwent revision surgery due to the obvious axial pain that lasted for two years. The complications of the two groups are shown in Table 5, and the difference was not statistically significant (*P* > 0.05). No serious complications such as spinal cord injury, wound and surgical site infection, recurrence of disc herniation, or epidural hematoma occurred in any of the patients.

## Discussion

CSS is the most common cause of spinal cord dysfunction in the middle-aged and elderly populations, with significant disability rates [24, 25]. The pathological basis of CSS is progressive compression of the spinal cord and nerve roots caused by cervical stenosis, leading to a series of neurological dysfunction symptoms due to ischemic changes in the nerves [1–3]. The clinical symptoms of CSS are complex, including myelopathy, radiculopathy, and myelo-radiculopathy [16, 26]. For CSS patients whose conservative treatments are ineffective or have progression of neurological symptoms, surgical intervention is usually recommended [27]. The main objectives of spine surgery are to relieve spinal cord compression, improve neurological function, maintain cervical sagittal sequence, correct deformities, and prevent further neurological deterioration.

ACDF is the standard, well-accepted open surgical procedure for treating CSS, with proven therapeutic efficacy [28, 29]. From an anterior cervical approach, ACDF can remove herniated disc tissue, posterior osteophytes, and calcified posterior longitudinal ligaments that compress the spinal cord and nerve roots without manipulating the spinal cord, thereby achieving adequate decompression of the spinal canal [30]. Furthermore, ACDF can also effectively correct cervical kyphosis deformity [31]. However, despite the many advantages of ACDF, complications can still occur. The anatomy of the anterior cervical spine is complex and variable, and prolonged intraoperative traction on structures such as the vascular sheath, trachea and esophagus is likely to lead to postoperative complications such as throat pain, hoarseness, and dysphagia [2, 6]. Dysphagia is the most common complication following anterior cervical spine surgery [16, 32], with a prevalence of ACDF ranging from 1.7 to 67% [32]. In addition, long-term complications of ACDF, such as pseudoarthrosis formation, implant nonunion, and instrumentation failure, are also important factors that can severely impair patient prognosis [2, 9]. The fusion rate of a single-level ACDF has been reported to reach approximately 92%, while the fusion rates for two-level and three-level ACDF have diminished success at 75% and 56%, respectively [30].

In recent years, with continuous innovation and refinement of minimally invasive endoscopic techniques, spinal endoscopy applied to lumbar degenerative diseases has matured, and has recently been gradually transitioning to the treatment of cervical degenerative diseases. Several studies have shown that minimally invasive endoscopic techniques in the cervical spine could be applied to treat CSS with good clinical outcomes [10, 15]. In a proof-of-concept in vitro trial, Eicker et al. [27] performed full-endoscopic arcocristectomy on 55 segments of cervical stenosis in 10 cadaveric specimens, resulting in an average increase of 4.1 mm (± 1.2 mm) in the sagittal diameter of the cervical canal postoperatively. The authors concluded that this technique for CSS was feasible, achieved sufficient decompression of the spinal canal, and preserved the integrity of most of the posterior structures. Currently, posterior cervical endoscopic techniques are becoming increasingly accepted and adopted. The posterior approach avoids the complex anatomical structures encountered with the anterior cervical approach, eliminating any related surgical complications, while sufficiently enlarging the spinal canal for successful decompression [6]. Additionally, compared with posterior open surgery, the posterior endoscopic technique does not require extensive removal of cervical bony tissue and extensive stripping of paravertebral muscles, which effectively avoids postoperative disruption of spinal stability and potential intractable neck and back pain caused by denervation of posterior cervical muscle groups [3, 33]. Studies have documented that maintenance of the normal sagittal sequence of the cervical spine following posterior surgery principally relies on the function of a dynamic system, incorporating muscles and ligaments, rather than bony fusion or other rigid structures [34]. Therefore, posterior cervical endoscopic surgery can ideally maintain the normal physiological state of the cervical spine while avoiding the possibility of postoperative cervical kyphosis deformity associated with posterior open surgery and adjacent segment degeneration caused by fusion and internal fixation.

In the present study, there were no significant differences between the two groups in A-VAS, N-VAS, JOA and NDI scores, and all postoperative patient clinical scores improved markedly compared with preoperative scores, meeting the clinical significance criteria of the MCID. Therefore, we concluded that both surgical techniques for the treatment of CSS achieved satisfactory clinical outcomes, significantly relieving patient pain and improving neurological function postoperatively. However, referring to the principal aim of the study, the endoscopic group had significantly superior results in terms of operative time, intraoperative blood loss, incision length, and hospital stay compared to ACDF, which indicated reduced operative trauma and quicker recovery, quite in line with the concept of enhanced recovery after surgery (ERAS). Notably, our team has extensively applied full endoscopic laminotomy decompression for the treatment of lumbar spinal stenosis in previous cases, accumulating substantial clinical experience [12]. The expertise has enabled us to facilitate more efficient reduction and maintenance of operative time when using this technique to treat CSS in the present study. Prolonged operative time is one of the essential risk factors contributing to increase the risk of surgical site infection [35]. The impact of surgery as documented on cervical spine imaging is closely related to patient prognosis, and spine stability and sagittal sequence balance affects cervical micromotor joint motion, which is key to postoperative symptom relief and maintenance. Fusion techniques can lead to reduced range of motion of the cervical vertebral body, which significantly increases stress loading on adjacent segments and accelerates the progression of disc degeneration [16, 36]. Postoperative ROM changes in adjacent segments in this study were less in the endoscopic group, whereas the ACDF group had a significantly increased change in ROM at 12 months postoperatively and at the last follow-up, with a statistically significant difference. In addition, cervical GROM was significantly reduced and cervical mobility was limited after ACDF due to internal fixation. There was a trend of decreasing disc signals in adjacent segments between the two groups, but the decrease in RVG was more pronounced in the ACDF group at the last follow-up, which may be mainly due to segmental hypermobility with excessive disc pressure.

The postoperative disc height was increased and was maintained at a good level in the ACDF group, whereas in the endoscopic group there was a gradual decrease in disc height over time. The change in disc height after endoscopic surgery was attributed to the natural degradation of the disc tissues as well as to disc degeneration aggravated by surgical disruption of the normal disc structure. These changes may lead to localized loss of cervical curvature and poor spinal alignment [16]. In addition, we did not observe any evidence of fracture of the lamina and facet joints or significant spondylolisthesis in the endoscopic group, and the ST was < 3 mm in all the patients. These results suggest that cervical segmental stability can remain well maintained throughout the follow-up period. Nevertheless, there was a trend of gradual increase in ST, which poses the possibility of cervical spine instability with decrease in disc height. Therefore, longer-term follow-up is still necessary to assess ultimate segmental stability. Notably, compared with the ACDF technique, endoscopic surgery confirmed better preservation of the disc structure. While this helps to maintain physiological mobility of the cervical spine, it could theoretically lead to an increased risk of recurrent disc herniation, especially when the annulus fibrosus structure is not intact. In the present study, we did not identify any cases of recurrence, with several possible explanations: cervical discs bear less load in body mechanics, endoscopic surgery has relatively limited effect on cervical spine biomechanics, or limited sample size of the study. Nevertheless, this potential complication is worthy of clinical attention.

All patients in the endoscopic group were successfully operated under local anesthesia without any case converted to open surgery. Regarding intraoperative complications in the endoscopic group, one neurological dysfunction case was attributed to prolonged intraoperative nerve root retraction, and two cerebrospinal fluid leakage cases were likely due to dense adhesion of osteophyte or ligamentum flavum at the posterior margin of the vertebral body to the dural sac, leading to a dural sac tear during the decompression procedure. In the ACDF group, there were two cases of axial pain or neurological dysfunction, two cases of cerebrospinal fluid leakage, one case each of dysphagia and hoarseness. There was no statistically significant difference in the complication rate between the two groups, and no serious complications such as spinal cord injury, wound and surgical site infection, recurrence of disc herniation, or epidural hematoma occurred in any of the patients.

Full endoscopic laminotomy decompression is a relatively new technique for the treatment of CSS, in which spinal stenosis segments and degree can be precisely targeted for decompression. This technique provides adequate decompression of the cervical spine with less surgical trauma than the standard open technique. From the results of this study, full endoscopic laminotomy decompression was demonstrated to have the following advantages over ACDF: (i) less surgical trauma, quicker recovery, and earlier postoperative functional exercises facilitating more rapid patient rehabilitation; (ii) under local anesthesia, endoscopic surgery allowed the surgeon to communicate with the patient intraoperatively, which helped to reduce the risk of injury to nerve roots and dural sac; in contrast, ACDF was performed under general anesthesia, which is associated with some risks and higher overall costs; (iii) spinal endoscopy was a safer surgical option for older patients who could not tolerate open surgery due to underlying medical conditions; (iv) endoscopic surgery provided a clear, magnified field of view that allowed for precise identification of structures such as the ligamentum flavum, nerve roots, dural sacs, and enabled precise decompression of target tissues, with less risk of injuring nerves and blood vessels; (v) endoscopic surgery for single-segment CSS eliminated the need for fusion and internal fixation, which preserved cervical motion segment mobility, and greatly reduced the impact of adjacent segment degeneration. However, cervical endoscopic techniques still have some limitations and obstacles, such as intraoperative difficulties, high technical requirements, and a long learning curve. In this study, local anesthesia is the preferred choice for CSS patients who underwent full endoscopic laminotomy decompression, but it necessitates a high level of operator proficiency and patient tolerance. This can be primarily attributed to the potential iatrogenic injury to nerve roots and/or spinal cord when neck movement occurs during the decompression process. The success of the procedure relies on effective collaboration between the surgeon and patient. Prior to surgery, patients should be informed about the possibility of a stress response in the neck due to decompression. Furthermore, patients were required to actively communicate with the surgeon of any discomfort during surgery. Importantly, surgeons must provide advance notice and exercise caution while decompressing areas (e.g., nerve roots, dural sacs) that may induce a stress response. Besides, it is inevitable that part of the vertebral plate and facet joints need to be removed during cervical endoscopy, which can lead to biomechanical changes of the cervical segments when improperly handled, and may accelerate degenerative changes of the cervical spine and vertebral instability in the long term. Previous studies have shown that stability of the cervical spine is put at significant risk when more than 50% of the facet joints are removed [37]. Raynor et al. [38] demonstrated in a cadaveric specimen experiment that a fracture occurred at 159 pounds of pressure when 70% of cervical facet joints were removed, whereas no fracture occurred at 208 pounds of pressure when only 50% of facet joints were removed. Therefore, to adequately enlarge the spinal canal for decompression while preserving the facet joints as much as possible is a key to the efficacy of cervical endoscopic surgery.

There are some limitations in the current study. Firstly, it is a retrospective study, which could not compare to one with double-blinding in the selection of surgical approaches, and was also liable to bias from subjective factors. Although we used PSM to minimize confounding factors between the two groups, some biases in the study results may still exist. Secondly, the sample size was relatively small, the follow-up period was short, and it was limited to a single center. Thirdly, although all imaging results were averaged over 3 measurements by 3 independent reviewers, measurement error could still exist.

## Conclusion

Full endoscopic laminotomy decompression is demonstrated to be an efficacious alternative technique to traditional ACDF for the treatment of single-segment CSS, with the advantages of less trauma, faster recovery, and less impact on cervical spine kinematics and adjacent segmental degeneration.

## Data Availability

The datasets used during the current study are available from the corresponding author on reasonable request.

## References

[1] Meyer F, Borm W, Thome C (2008). Degenerative cervical spinal stenosis: current strategies in diagnosis and treatment. Dtsch Arztebl Int.

[2] Jiang Y, Li X, Zhou X (2017). A prospective randomized trial comparing anterior cervical discectomy and fusion versus plate-only open-door laminoplasty for the treatment of spinal stenosis in degenerative diseases. Eur Spine J.

[3] Tong Y, Huang Z, Hu C (2020). A comparison study of posterior cervical percutaneous endoscopic ventral bony decompression and simple dorsal decompression treatment in cervical spondylotic radiculopathy caused by cervical foraminal and/or lateral spinal stenosis: a clinical retrospective study. BMC Musculoskelet Dis.

[4] Ghogawala Z, Terrin N, Dunbar MR (2021). Effect of ventral vs dorsal spinal surgery on patient-reported physical functioning in patients with cervical spondylotic myelopathy. JAMA.

[5] Kong W, Xin Z, Du Q, Cao G, Liao W (2019). Anterior percutaneous full-endoscopic transcorporeal decompression of the spinal cord for single-segment cervical spondylotic myelopathy: the technical interpretation and 2 years of clinical follow-up. J Orthop Surg Res.

[6] Yuan H, Zhang X, Zhang L, Yan Y, Liu Y, Lewandrowski K (2020). Comparative study of curative effect of spinal endoscopic surgery and anterior cervical decompression for cervical spondylotic myelopathy. J Spine Surg.

[7] Bakhsheshian J, Mehta VA, Liu JC (2017). Current diagnosis and management of cervical spondylotic myelopathy. Glob Spine J.

[8] Lee NJ, Kim JS, Park P, Riew KD (2022). A comparison of various surgical treatments for degenerative cervical myelopathy: a propensity score matched analysis. Glob Spine J.

[9] Mesregah MK, Formanek B, Liu JC, Buser Z, Wang JC (2023). Perioperative complications of surgery for degenerative cervical myelopathy: a comparison between 3 procedures. Glob Spine J.

[10] Lv J, Mei J, Feng X, Tian X, Sun L (2022). Clinical efficacy and safety of posterior minimally invasive surgery in cervical spondylosis: a systematic review. J Orthop Surg Res.

[11] Zhang C, Wu J, Zheng W, Li C, Zhou Y (2020). Posterior endoscopic cervical decompression: review and technical note. Neurospine.

[12] Jiang Q, Ding Y, Lu Z (2023). Comparative analysis of non-full and full endoscopic spine technique via interlaminar approach for the treatment of degenerative lumbar spinal stenosis: a retrospective, single institute, propensity score-matched study. Glob Spine J.

[13] Parker SL, Godil SS, Shau DN, Mendenhall SK, Mcgirt MJ (2013). Assessment of the minimum clinically important difference in pain, disability, and quality of life after anterior cervical discectomy and fusion: clinical article. J Neurosurg Spine.

[14] Kato S, Oshima Y, Matsubayashi Y, Taniguchi Y, Tanaka S, Takeshita K (2019). Minimum clinically important difference and patient acceptable symptom state of Japanese orthopaedic association score in degenerative cervical myelopathy patients. Spine.

[15] Ahn Y, Keum HJ, Shin SH (2020). Percutaneous endoscopic cervical discectomy versus anterior cervical discectomy and fusion: a comparative cohort study with a five-year follow-up. J Clin Med.

[16] Zhang J, Ruan D, Xuan A (2023). Comparative study of outcomes between allograft intervertebral disc transplantation and anterior cervical discectomy and fusion: a retrospective cohort study at least 5 years of follow-up. Eur Spine J.

[17] Obo T, Fujishiro T, Mizutani M (2022). Segmental cervical instability does not drive the loss of cervical lordosis after laminoplasty in patients with cervical spondylotic myelopathy. Spine J.

[18] Ito M, Tadano S, Kaneda K (1993). A biomechanical definition of spinal segmental instability taking personal and disc level differences into account. Spine.

[19] Limanówka B, Sagan L (2020). Changes in cervical range of motion following anterior cervical discectomy with fusion—preliminary results. Neurol Neurochir Pol.

[20] Schneiderman G, Flannigan B, Kingston S, Thomas J, Dillin WH, Watkins RG (1987). Magnetic resonance imaging in the diagnosis of disc degeneration: correlation with discography. Spine.

[21] Badhiwala JH, Witiw CD, Nassiri F (2019). Efficacy and safety of surgery for mild degenerative cervical myelopathy: results of the aospine North America and international prospective multicenter studies. Neurosurgery.

[22] Bonosi L, Musso S, Cusimano LM (2023). The role of neuronal plasticity in cervical spondylotic myelopathy surgery: functional assessment and prognostic implication. Neurosurg Rev.

[23] Kadoya S (1992). Grading and scoring system for neurological function in degenerative cervical spine disease–neurosurgical cervical spine scale. Neurol Med Chir.

[24] Sangondimath G, Mallepally AR, Marathe N, Mak K, Salimath S (2020). Degenerative cervical myelopathy: recent updates and future directions. J Clin Orthop Trauma.

[25] Manchikanti L, Malla Y, Cash KA, Mcmanus CD, Pampati V (2012). Fluoroscopic epidural injections in cervical spinal stenosis: preliminary results of a randomized, double-blind, active control trial. Pain Phys.

[26] Meng Z, Yu J, Luo C (2017). Anterior cervical spondylosis surgical interventions are associated with improved lordosis and neurological outcomes at latest follow up: a meta-analysis. Sci Rep.

[27] Eicker SO, Klingenhöfer M, Stummer W, Steiger H, Hänggi D (2012). Full-endoscopic cervical arcocristectomy for the treatment of spinal stenosis: results of a cadaver study. Eur Spine J.

[28] Ambati VS, Macki M, Chan AK (2023). Three-level acdf versus 3-level laminectomy and fusion: are there differences in outcomes? An analysis of the quality outcomes database cervical spondylotic myelopathy cohort. Neurosurg Focus.

[29] Landers MR, Addis KA, Longhurst JK, Vom Steeg B, Puentedura EJ, Daubs MD (2013). Anterior cervical decompression and fusion on neck range of motion, pain, and function: a prospective analysis. Spine J.

[30] Choi SH, Kang C (2020). Degenerative cervical myelopathy: pathophysiology and current treatment strategies. Asian Spine J.

[31] Chen SR, Levasseur CM, Pitcairn S (2021). Surgery-related factors do not affect short-term adjacent segment kinematics after anterior cervical arthrodesis. Spine.

[32] Epstein NE (2019). A review of complication rates for anterior cervical diskectomy and fusion (ACDF). Surg Neurol Int.

[33] Li C, Tang X, Chen S, Meng Y, Zhang W (2019). Clinical application of large channel endoscopic decompression in posterior cervical spine disorders. BMC Musculoskelet Dis.

[34] Nolan JJ, Sherk HH (1988). Biomechanical evaluation of the extensor musculature of the cervical spine. Spine.

[35] Cheng H, Chen BP, Soleas IM, Ferko NC, Cameron CG, Hinoul P (2017). Prolonged operative duration increases risk of surgical site infections: a systematic review. Surg Infect.

[36] Eck JC, Humphreys SC, Lim TH (2002). Biomechanical study on the effect of cervical spine fusion on adjacent-level intradiscal pressure and segmental motion. Spine.

[37] Zdeblick TA, Zou D, Warden KE, Mccabe R, Kunz D, Vanderby R (1992). Cervical stability after foraminotomy: a biomechanical in vitro analysis. J Bone Jt Surg Am.

[38] Raynor RB, Pugh J, Shapiro I (1985). Cervical facetectomy and its effect on spine strength. J Neurosurg.

